# Profiling the clinical presentation of diagnostic characteristics of a sample of symptomatic TMD patients

**DOI:** 10.1186/1472-6831-12-26

**Published:** 2012-08-02

**Authors:** Luciana Pimenta e Silva Machado, Marianita Batista de Macedo Nery, Cláudio de Góis Nery, Cláudio Rodrigues Leles

**Affiliations:** 1 School of Dentistry, Federal University of Goias, Goiania, Goias, Brazil; 2 Private practice, Goiania, Goias, Brazil; 3 DPRO/Faculdade de Odontologia, Praça Universitária, s/n. Setor Universitário, CEP 74.605-220, Goiania, Goias, Brazil

**Keywords:** Temporomandibular disorders, Cluster analysis, Clinical diagnosis, Diagnosis-related groups

## Abstract

**Background:**

Temporomandibular disorder (TMD) patients might present a number of concurrent clinical diagnoses that may be clustered according to their similarity. Profiling patients’ clinical presentations can be useful for better understanding the behavior of TMD and for providing appropriate treatment planning. The aim of this study was to simultaneously classify symptomatic patients diagnosed with a variety of subtypes of TMD into homogenous groups based on their clinical presentation and occurrence of comorbidities.

**Methods:**

Clinical records of 357 consecutive TMD patients seeking treatment in a private specialized clinic were included in the study sample. Patients presenting multiple subtypes of TMD diagnosed simultaneously were categorized according to the AAOP criteria. Descriptive statistics and two-step cluster analysis were used to characterize the clinical presentation of these patients based on the primary and secondary clinical diagnoses.

**Results:**

The most common diagnoses were localized masticatory muscle pain (n = 125) and disc displacement without reduction (n = 104). Comorbidity was identified in 288 patients. The automatic selection of an optimal number of clusters included 100% of cases, generating an initial 6-cluster solution and a final 4-cluster solution. The interpretation of within-group ranking of the importance of variables in the clustering solutions resulted in the following characterization of clusters: chronic facial pain (n = 36), acute muscle pain (n = 125), acute articular pain (n = 75) and chronic articular impairment (n = 121).

**Conclusion:**

Subgroups of acute and chronic TMD patients seeking treatment can be identified using clustering methods to provide a better understanding of the clinical presentation of TMD when multiple diagnosis are present. Classifying patients into identifiable symptomatic profiles would help clinicians to estimate how common a disorder is within a population of TMD patients and understand the probability of certain pattern of clinical complaints.

## Background

Temporomandibular disorders (TMD) are a group of painful conditions that typically involve the temporomandibular joint(s) (TMJ) and/or masticatory muscles [1]. It is well recognized that a proper diagnosis is essential for the successful treatment planning of TMD and that this is one of the greatest challenges facing the professionals who treat these conditions. The multifactorial aetiology, the similar clinical manifestations and overlapping of the multiple signs and symptoms of TMD makes its clinical management a complex task to overcome [1,2].

It is clear from clinical practice that TMD and orofacial pain patients share many common clinical features or symptoms. For instance, patients with chronic muscle pain associated with long-term parafunctional habits, such as clenching or bruxism, may develop secondary structural changes in the temporomandibular joints (or central excitatory effects leading to myofascial muscle pain [1,3]).

A particular profile of patients seeking treatment for these conditions could be composed by reflecting the heterogeneity of the conditions involved into identifiable groups based on the clinical presentation of symptomatic patients. The classification of groups of patients who share properties in common might provide useful information for the diagnosis and initial management of TMD. The aim of this study was to classify the symptom presentation of patients who sought treatment and who were diagnosed with a variety of combined subtypes of TMD and to cluster them into homogenous groups based on their clinical condition and chief complaints.

## Methods

The study was a retrospective assessment of the clinical records of 357 consecutive symptomatic patients, 86.8% female, age ranging from 11 to 70 years old (mean = 31.9; SD = 11.1), referred to or seeking treatment for orofacial pain and/or TMD in a private clinic in Goiania, Goias, Brazil. Patients’ characteristics, inclusion and exclusion criteria and information about clinical examination and data collection were reported in a previous study [4]. The study protocol was approved by the Ethical Research Committee of the Clinical Hospital of the Federal University of Goias.

Anamnesis and all clinical examination were performed by two trained dentists with a large amount of experience in dealing with patients with orofacial pain as part of the routine clinical practice of the private clinic. Both examiners were trained and certified in the same Orofacial Pain Center (University of Kentucky, USA). Standardized criteria of the AAOP were used in all clinical assessment and diagnosis.

The information collected from patients during anamnesis included their chief complaint and other secondary complaints, if present, the patients’ reports of their perceptions about their pain status, and their dental and medical histories. It also included questions about pain characteristics (onset, duration, frequency, quality, pain score on the Visual Analogue Scale and aggravating or relieving factors, amongst others), a description of the symptoms experienced since the onset of pain (such as earache, dizziness, decrease of mouth opening, nausea/vomiting and weakness in the masticatory muscles, among others), headache and related characteristics, the presence of perceived joint noises, the history of locking and/or trauma in the orofacial region, the presence of conscious oral habits (clenching, nail biting, gum chewing and putting the phone between the ears and shoulder, among others), the characteristics of sleep and diet, the type of professionals previously sought and any previous treatments. The questions for pain assessment were planned to get a comprehensive clinical evaluation for diagnostic and treatment purposes. It included opened questions about the features of the pain sensation, and the visual analogue scale was used to measure subjective characteristics or attitudes that cannot be directly measured, such as pain intensity (how intensely individuals are feeling pain), and to monitor the effectiveness of subsequent treatments. Sleep characteristics were assessed in the initial appointment using general opened questions such as *“Do you think you sleep well?”, “How many hours do you sleep per night?”, “How long it takes to sleep?”, “How many times you wake during the night?”, “Your sleep restores your energy?”, “Do you feel rested when you wake in the morning?”.* Some patients with suspect of severe sleep disorders were diagnosed by polissomnography.

The physical examination included cranial nerve functioning, cervical movement pain or limitation, palpation of masticatory and cervical muscles, a functional examination of the masticatory muscles with muscle stimulation tests and an evaluation of the range of mandibular motion. Additional exams and sectional images of the TMJ were requested and performed when needed, in cases where internal TMJ disorders were suspected.

The American Academy of Orofacial Pain (AAOP) guidelines and diagnostic criteria [1] were adopted for diagnosing the patients. Diagnostic subgroups were classified into muscular disorders (local masticatory muscle pain, masticatory myofascial pain, protective co-contraction, myospasm and tendinitis), articular disorders (disc displacement with reduction, disc displacement without reduction and subluxation), inflammatory TMJ disorders (synovitis/capsulitis), non-inflammatory TMJ disorders (primary and secondary osteoarthritis), cervical muscle disorders (local cervical pain, cervical myofascial pain) and bruxism.

The diagnosis of internal TMJ disorders was based on clinical findings and conventional radiographs when indicated. Magnetic resonance images of the TMJ were requested for patients with persistent pain, significant limitations in mouth opening (< 30 mm) and suspected degenerative joint disease. Although bruxism is a contributor and might occasionally trigger TMD, it was considered a diagnostic group in cases when it was a chief complaint.

When patients presented with more than one TMD, both the main and the secondary diagnosis were registered. The main diagnosis was based on the chief complaint reported by the patient and the secondary diagnosis was based on other relevant complaints or significant clinical findings elicited by clinical examination or the imaging methods. All other possible, additional diagnoses were registered but not considered for the characterization of the patients’ symptomatic profiles. Patients with tension-type headache, migraine, neuropathic pain and sleep disorder identified as the only diagnosis were excluded from the sample, except when primary headache was caused by a TMD or was triggered by muscle function or TMJ disorders. In such cases, for the purpose of the cluster analysis, only the TMD was considered for analysis. A complete description of the frequencies of patients’ diagnoses was published elsewhere [4].

Data were analysed using a descriptive analysis and the non-hierarchical two-step cluster analysis procedure as an exploratory tool intended to reveal natural groupings (or clusters) within the data set that would otherwise not be apparent. The frequency analysis included the primary and secondary diagnoses and a cross-tabulation of the combined diagnoses. In the next stage, the two-step cluster analysis was used to divide samples into *n* numbers of clusters based on the primary and secondary diagnoses using an auto-clustering algorithm to reach an initial clustering solution. All TMDs were inserted in the cluster analysis as yes/no dichotomous variables. The relative importance of variables (diagnoses) with statistical significance in the formation of clusters (chi-square test) helped to identify the occurrence of yes/no responses to the different diagnoses. The frequencies of both positive (presence) and negative (absence) diagnoses with their statistical significance were considered for the identification of variables that contributed most to the differentiation of clusters.

The initial cluster solution was used to identify relevant variables for interpreting the groups. Alternative solutions, other than the default auto-clustering option, were tried to disclose fewer natural groupings using a specific and fixed number of clusters. The proposed clustering solutions were selected according to clinical interpretability and plausibility. Finally, all clusters were named using a term that best represented the patients’ symptomatic characteristics, according to the combination of the primary and secondary TMD diagnoses.

The SPSS 17.0 software (SPSS Inc., Chicago, IL, USA) was used for all statistical analyses.

## Results

Table 1 shows the absolute frequency of primary and secondary TMD diagnoses. The most common diagnosis was localized masticatory muscle pain (LMP) in 125 patients (35.0%), followed by disc displacement without reduction (DDWOR) in 104 patients (29.1%). A secondary diagnosis was identified in 288 patients (80.7%). The combined diagnoses are described in Table 2. They showed great diversification in the original symptomatic characteristics of the patients and that only 23 (6.4%) had a primary diagnosis alone.

**Table 1:** Frequency of primary and secondary diagnostic classifications of symptomatic patients

**TM disorders and abbreviations**		**Primary**	**Secondary**	**Total**
		**diagnosis**	**diagnosis**	
		**(n = 357)**	**(n = 288)**	
Localized masticatory muscle pain	LMP	88	37	125
Disc displacement without reduction	DDWOR	15	89	104
Capsulitis/synovitis	Cap/Syn	75	17	92
Cervical myofascial pain	CMP	53	27	80
Secondary osteoarthrosis	SOA	3	48	51
Disc displacement with reduction	DDWR	36	9	45
Masticatory myofascial pain	MMP	37	6	43
Bruxism	Brux	5	37	42
Tendinitis	Tend	19	6	25
Primary osteoarthrosis	POA	9	2	11
Myospasm	Myosp	6	2	8
Localized cervical pain	LCP	5	3	8
Protective co-contraction	PCC	3	5	8
Subluxation	Sublux	3	0	3

**Table 2:** Cross-tabulation of combined primary and secondary diagnoses in TMD symptomatic patients

	**Secondary diagnosis**															
**Primary diagnosis**	**None**	**LMP**	**Cap/Syn**	**CMP**	**MMP**	**DDWR**	**Tend**	**DDWOR**	**POA**	**LCP**	**Myosp**	**PCC**	**Sublux**	**SOA**	**Brux**	**Total**
LMP	3		7	23	1	3	2				1			16	15	71
Cap/Syn	10	7		1	3	3		37	1	3		2			3	70
CMP		23	2			2	1	21						2		51
MMP			5	1		1		4				1		7	17	36
DDWR	2	1	2		2		3	8			1	2		6	1	28
Tend		4						7	1					3	1	16
DDWOR	7	1												6		14
POA				1				1						5		7
LCP			1					3						1		5
Myosp		1		1				3								5
PCC								1						1		2
Sublux								2								2
SOA	1							1								2
Brux								1						1		2
Total	23	37	17	27	6	9	6	89	2	3	2	5	0	48	37	311

Following the frequency analysis, the two-step cluster analysis procedure was to disclose the natural groupings. An initial solution with six clusters was achieved using the auto-clustering algorithm. Table 3 shows the data revealing which variables (or TMD diagnoses) were important in the formation of clusters, retrieved from the cluster charts and showing the category frequency by cluster. The chi-square test was used to measure the importance of the variables in each cluster, sorted by the importance ranking of each variable.

**Table 3:** Relative importance of variables with statistical significance in the formation of clusters, ranked by chi-square test values

**Cluster**	**n**	**Variable**	**Frequency**		Chi- square*
			**Yes**	**No**	
I	72	LMP	65	7	99.4
		DDWOR	-	72	26.5
		Brux	22	50	24.5
		CMP	-	72	20.8
		DDWR	-	72	10.4
		MMP	-	72	9.9
				
II	83	DDWR	37	46	77.0
		Tend	25	58	68.0
		Cap/Syn	3	80	21.3
		POA	9	74	16.7
		LMP	11	72	16.5
		DDWOR	7	76	14.4
		MMP	-	83	11.4
III	35	MMP	35	-	255.6
		Brux	17	18	45.7
		LMP	1	34	15.5
		Cap/Syn	-	35	12.2
IV	46	CMP	46	-	159.3
		LMP	46	-	87.5
		DDWOR	-	46	16.9
		Cap/Syn	-	46	16.0
V	72	Cap/Syn	72	-	207.4
		LMP	-	72	37.8
		DDWOR	37	35	22.0
		CMP	-	72	20.8
		SOA	-	72	12.0
		Brux	-	72	9.6
VI	49	DDWOR	48	-	125.9
		LMP	-	49	25.8
		Cap/Syn	-	49	17.0
		Sublux	3	46	16.4
		CMP	21	28	11.8

* All Chi-square values were p < 0.001.

The combined interpretation of Table 3 (ranking of variable importance) and Table 4 (proportion of primary diagnoses) revealed that Cluster n#1 (n = 72) was characterized by a high prevalence of LMP as the primary diagnosis, the absence of internal derangement (DDWOR and DDWR) and myofascial pain (CMP and MMP), and a high prevalence of bruxism as the secondary diagnoses. Cluster n#2 (n = 83) mainly included patients with acute internal derangements (DDWR and/or tendinitis and/or primary osteoarthosis) and a low frequency of muscular diagnoses. Cluster n#3 (n = 35) was almost exclusively composed of patients with masticatory myofascial pain as the primary diagnosis, and other relevant features such as bruxism as the secondary diagnosis, and the absence of articular disorders.

**Table 4:** Frequency of TMD diagnoses from initial cluster solutions with relevant variables for the interpretation of clusters

**Cluster**	**(n)**	**Frequency of TMD diagnoses (frequency of primary diagnoses in parentheses)**											**Relevant variables for interpretation of clusters**
		**LMP**	**DDWOR**	**DDWR**	**Tend**	**MMP**	**CMP**	**Cap/Syn**	**Brux**	**POA**	**SOA**	**Sublux**	
I	72	65 (58)	0	0	*	0	0	*	22 (4)	*	*	*	LMP
II	83	11 (6)	7 (0)	37 (32)	25 (19)	0	*	3 (1)	-	9 (8)	*	*	DDWR + Tend
III	35	1 (1)	*	*	*	35 (32)	*	0	17 (0)	*	*	*	MMP
IV	46	46 (23)	0	*	*	*	46 (23)	0	*	*	*	*	LMP + CMP
V	72	0	37 (0)	*	*	*	0	72 (64)	0	*	0	*	Cap/Syn + DDWOR
VI	49	0	48 (15)	*	*	*	21 (21)	0	*	*	*	3 (3)	DDWOR + CMP
Total	357	123 (88)	92 (15)	37 (32)	25 (19)	35 (32)	67 (44)	75 (65)	39 (4)	9 (8)	0	3 (3)	-

* Variable not important in the formation of clusters.

Patients with acute cervical and/or localized masticatory muscle pain, without any articular complaints, comprised cluster n#4 (n = 46). Conversely, cluster n#5 (n = 72) was mainly composed of patients with acute articular pain (capsulitis/synovitis) and secondary DDWOR. Cluster n#6 included patients with cervical pain, non-painful chronic articular impairment (DDWOR) and a history of TMJ subluxation.

**Figure 1:**
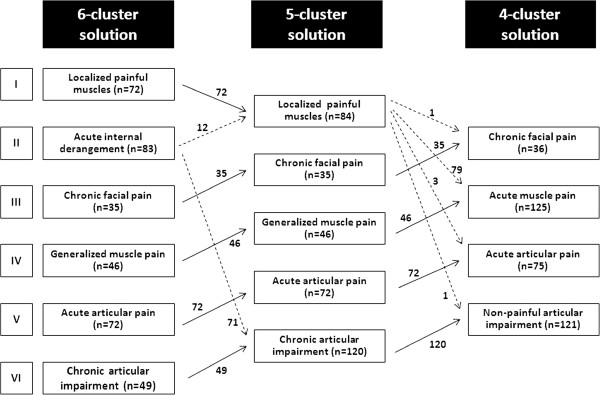
Initial and alternative cluster solutions for interpretation of the patients’ symptomatic profiles.

Figure 1 shows the range of solutions with alternative 5- and 4-cluster solutions. In the initial 6-cluster solution, the clusters were named as follows: (1) localized painful muscles, (2) acute internal derangement, (3) chronic facial pain, (4) generalized muscle pain, (5) acute articular pain, and (6) chronic articular impairment. Of the two solutions with fewer groups, the cases were rearranged and the results were reinterpreted in the final 4-cluster solution as follows: (1) chronic facial pain, (2) acute muscle pain, (3) acute articular pain, and (4) non-painful articular impairment. The prevalence of the patients’ symptomatic profiles were 10.1% for chronic facial pain, 35.0% for acute muscle pain, 21.0% for acute articular pain and 33.9% for non-painful articular impairment.

## Discussion

The results of this study present different symptomatic profiles of patients seeking treatment in a TMD clinic, who were classified by cluster analysis into four to six homogenous groups based on their clinical presentation.

It is important to note that the cluster names were intuitively defined considering the time of onset, the location, the presence of pain and extent of the symptoms. Patients’ profiles may help clinicians to quickly recognize patient characteristics and provide a reasonable treatment approach. After clustering procedures, data also illustrate the prevalence of symptomatic profiles, which is helpful for identification of symptomatic profiles, would help clinicians to estimate how common a disorder is within a population of TMD patients and understand the probability of certain pattern of clinical complaints.

Although internal validity of the diagnostic criteria and the efficacy of the examiners’ performance were not tested, standardized criteria of the AAOP were used and both examiners were trained and certified in the same Orofacial Pain Center (University of Kentucky, USA).

Masticatory muscle pain is recognized as the most common complaint in patients in general practice, and perhaps only a few patients will present for treatment in private practice with a chronic stage. This could be explained by the fact that many patients might have previously searched for treatments that turned out to be ineffective and then began to give up on seeking new alternatives, leading to an even more chronic problem [5], or it could be because many muscle or joint disorders do not only go unnoticed by patients and professionals in general practice, but also because most professionals do not know how to treat them properly. All those situations may worsen the problem [5-7].

In particular, this could occur with some internal derangement of the TMJ, which will eventually become a chronic condition quite easily. Kalaykova et al. [8] studied the natural course of patients with anterior disc displacement with reduction after two years and observed that clicking commonly disappear completely without symptoms of permanent locking even with the disc displacement still present, but with no, or only a partial, reduction. They also observed that intermittent locking may be indicative of the development of a disc displacement without reduction which is only rarely accompanied by symptoms of permanent locking [8].

These could be some of the reasons why patients with acute muscle pain and chronic joint impairments were more frequent in this sample. Many patients do not know what to do in these situations or which professionals they should look for: an orthodontist, a maxillofacial surgeon, a prosthodontist or an otolaryngologist, for example. Moreover, many professionals are unsure about what to say to patients with this distressing disorder, when (or whether) to refer them to specialists and even which experts to refer them to [7].

Another explanation for the high frequency of acute problems could be the fact that most of the TMDs are cyclical. Patients with a chronic condition that they had already been adapted, for some reason or event, may have worsened and make them seek for treatment [1,9].

A strong relationship between sore muscles (myalgia or localized masticatory muscle pain) and bruxism (patients from cluster 2), myofascial masticatory muscle pain and bruxism (patients from cluster 1) and the need to seek for treatment was revealed by our results. A similar finding was found by Lobbezzo-Scholte et al. [10], whose results showed relatively more patients of the mainly myogenous component group who reported clenching and grinding than the other patient groups. Although a relationship is not clear from the literature, one could suppose that bruxism may be highly associated with pain referred from masticatory muscles [11], initiating or perpetuating masticatory muscle soreness or pain [12-14].

The significance of dealing with bruxism and masticatory muscle pain early on and the importance of recognizing these conditions in everyday practice are highlighted, since myalgia, being a deep pain stimulus, can yield central excitatory effects and referred pain. Therefore, this statement may explain the fact that when a muscle pain is maintained, more muscle pain may be restarted, keeping the patient in a cycle of pain [9]. This entire situation might be sustained by bruxism (clenching or grinding) as an initiating or perpetuating factor for muscle disorders [13,14].

A treatment plan could be outlined for patients with similar characteristics as for those comprising clusters 1 and 2 with muscle dysfunctions and bruxism. A more conservative management should be carried out since such disorders are related to multiple factors, and often, if not always, there is a strong role of emotional factors [2,9,12,15-18]. This might include pharmacological therapy, cognitive-behavioural therapies, psychotherapy, self-care (resting, relaxation techniques, massage, hot and/or cold packs, stretching or exercise), physiotherapy (jaw exercises, postural training), low-level laser therapy and wearing occlusal appliances [19-27].

A combination of capsulitis/synovitis and disc dislocation without reduction (DDWOR) was found in patients from cluster 3. Regarding the concept that internal TMJ derangement is significantly involved in the production of TMJ pain and dysfunction, these results corroborate findings from other authors [28]. These findings may also strengthen the idea that patients with a primary TMJ pathology, such as a DDWOR, frequently develop an inflammatory response to the dysfunctional disc-condyle relationship, clinically represented by a diagnosis of capsulitis/synovitis [3,9,29]. While DDWOR can be seen as a chronic disorder, is quite evident here that acute pain caused by capsulitis/synovitis may be the main reason why patients seek specialized treatment. The first efforts in treating patients with similar characteristics should be directed towards the complaint of pain: the reason why the patient initially sought treatment. In the case of an acute DDWOR, an attempt should be made to unlock the patient [30]. While most therapies are not evidence-based, we strongly recommend a conservative approach, which may include patient orientation, pharmacotherapy, physical therapy for pain (thermotherapy, low-level laser therapy) and then physical therapy to improve function (passive TMJ manipulation) and the use of occlusal appliances [9,26,30].

Patients presenting with non-painful articular impairment from cluster 4 were the second most frequent group of patients who were referred for or who sought treatment for TMD. Despite the fact that this cluster was named TMJ impairments, due to the main disorders clustered, perhaps we can infer that treatment seeking was mainly guided by dysfunctions associated with muscle disorders or DDWR, as shown in Table 4.

As discussed above for clusters 1 to 3, a combination of treatment modalities can also be outlined for the management of patients who have a similar presentation to those of cluster 4, due to the many associated muscle and joint disorders. Each particular case should be evaluated in order to define appropriate treatment planning. As there are many therapeutic options, if the selected treatment does not eliminate the patient’s pain complaint, the next more complex condition or chronic muscle condition should be considered by the clinician [31]. Due to the chronicity of the clinical conditions presented by these patients, a psychosocial component (such as anxiety and depression) may be present and thus a psychological approach should be included in the treatment plan [9,31,32].

Velly et al. [12] clustered 162 patients with TMD based on their clinical condition and degree of severity, and they also studied some related factors such as psychosocial aspects. Although the authors also obtained four clusters of TMD patients (two clusters with more than one TMD condition and two with only one), the profiles of patients in the clusters obtained were different from the clusters found in this sample; one of the reasons for this was the diagnostic criteria adopted by these authors—the Research Diagnostic Criteria for Temporomandibular Disorders (RDC/TMD) [33]. In our study, emotional and psychosocial aspects would be either cause or effect of the TM disorders. They were only addressed as part of the overall treatment approach and were not included in this study. We consider that our study used routinely collected data that did not describe in full detail other variables that are associated with the cause or the effect of TMDs. Cross-sectional studies using data originally collected for other purposes are often unable to include data on confounding factors or other variables that affect the relationship between the presumed cause and effect. This may be viewed as a weakness of the study design and, certainly, a consequence of the limited scope of our study since we decided to focus on the description of the distribution of patients’ common symptomatic profiles.

Despite some limitations regarding the generalization of results (better related to patients seeking treatment, cases derived from only one private clinic and the evaluation and classification system adopted did not contemplate the RDC/TMD), we believe that the most interesting aspects that deserve particular attention from the results of our study are the large sample size, the large number of patients presenting with more than one TMD condition simultaneously diagnosed and the strong homogeneous relationship between patients in the formation of clusters. The clustering method may depend on the choice of classification variables and how they were collected [12]. However, bias was avoided in the cluster formation by use of the two-step cluster analysis, because it does not involve hypothesis testing or a pre-determined number of clusters, perfectly acceptable for cluster data that may not meet the assumptions for best performance. As a non-hierarchical method of clustering, the two-step cluster method has two advantages over the hierarchical methods used in similar studies: it is less impacted by outlier elements and the final solution optimizes within-cluster homogeneity and between-cluster heterogeneity [34]. Finally, identifying the characteristics of each group of patients seeking treatment may direct future research in the pursuit of outcomes at a higher level of efficacy and allow a more appropriate clinical decision-making process for patients with multiple, simultaneous TMDs.

## Conclusion

In summary, the great homogeneity of patients with multiple conditions of TMD being diagnosed while seeking treatment allowed the recognition of subgroups of conditions. Determining the profile of patients with such characteristics is of paramount importance for understanding their clinical presentation and thus delineating the most appropriate treatment for them. The identification of these patients’ profiles and estimation of their prevalence within TMD symptomatic patients seeking treatment may be useful for the management of patients seeking treatment and for reducing misleading clinical decisions due to the large heterogeneity of TMD diagnostic subgroups.

## Competing interests

The authors declare that they have no competing interests.

## Authors’ contributions

LPSM performed all data collection, created the database and drafted the manuscript, MBMN and CGN performed all clinical examinations, and CRL was the head of the study, performed the statistical analysis and collaborated in manuscript design and writing. All authors read and approved the final manuscript.

## Pre-publication history

The pre-publication history for this paper can be accessed here:

http://www.biomedcentral.com/1472-6831/12/26/prepub
